# Development of a Mucus Gland Bioreactor in Loach *Paramisgurnus dabryanus*

**DOI:** 10.3390/ijms22020687

**Published:** 2021-01-12

**Authors:** Tong Zhou, Bolan Zhou, Yasong Zhao, Qing Li, Guili Song, Zuoyan Zhu, Yong Long, Zongbin Cui

**Affiliations:** 1 State Key Laboratory of Freshwater Ecology and Biotechnology, Institute of Hydrobiology, Chinese Academy of Sciences, Wuhan 430072, China; TongZhou_2020@163.com (T.Z.); zhoubolan198769@163.com (B.Z.); mz20150908@163.com (Y.Z.); qli@ihb.ac.cn (Q.L.); guilisong@ihb.ac.cn (G.S.); zyzhu@ihb.an.cn (Z.Z.); 2 College of Advanced Agricultural Sciences, University of Chinese Academy of Sciences, Beijing 100049, China; 3 Guangdong Provincial Key Laboratory of Microbial Culture Collection and Application, State Key Laboratory of Applied Microbiology Southern China, Guangdong Institute of Microbiology, Guangdong Academy of Sciences, Guangzhou 510070, China

**Keywords:** bioreactor, loach, transgenic animal, mucus gland, interferon

## Abstract

Most currently available bioreactors have some defects in the expression, activity, or purification of target protein and peptide molecules, whereas the mucus gland of fish can overcome these defects to become a novel bioreactor for the biopharmaceutical industry. In this study, we have evaluated the practicability of developing a mucus gland bioreactor in loach (*Paramisgurnus dabryanus*). A transgenic construct pT2-krt8-IFN1 was obtained by subcloning the promoter of zebrafish keratin 8 gene and the type I interferon (IFN1) cDNA of grass carp into the SB transposon. The IFN1 expressed in CIK cells exhibited an antiviral activity against the replication of GCRV873 and activated two genes downstream of JAK-STAT signaling pathway. A transgenic loach line was then generated by microinjection of the pT2-krt8-IFN1 plasmids and in vitro synthesized capped SB11 mRNA. Southern blots indicated that a single copy of IFN1 gene was stably integrated into the genome of transgenic loach. The expression of grass carp IFN1 in transgenic loaches was detected with RT-PCR and Western blots. About 0.0825 µg of grass carp IFN1 was detected in 20 µL mucus from transgenic loaches. At a viral titer of 1 × 10^3^ PFU/mL, plaque numbers on plates containing mucus from transgenic loaches reduced by 18% in comparison with those of the control, indicating that mucus of IFN1-transgenic loaches exhibited an antiviral activity. Thus, we have successfully created a mucus gland bioreactor that has great potential for the production of various proteins and peptides.

## 1. Introduction

Protein and peptide drugs are playing important roles in protection of human and animals from various diseases and their market demands are quickly increased year by year (http://www.bccresearch.com). The transgenic bioreactor can be developed by the introduction of a protein-encoding gene under the control of a promoter into the genome of microorganisms, plants or animals to effectively produce target proteins and peptide molecules with biological activities of economic values.

Currently available bioreactors, such as bacteria [1], yeasts [2], transgenic plants [3], and transgenic animals [4], have produced a number of drugs that are widely used for medical and experimental purposes, but these bioreactors have to overcome some defects such as a lack of post-translational modification for biological activities of target proteins and peptides, high costs for the generation of bioreactors and purification of target proteins and peptides, or products that are toxic to bioreactors [5,6].

Fish is considered as the most suitable market-oriented transgenic animal for human consumption [7]. With the successful development of the first transgenic fish [8], more than 35 species of fish have been used for gene transfer studies to improve growth rate, disease resistance and antifreeze and to dissect gene functions. Fish exhibit some advantages over other organisms for development of bioreactors, such as short breeding cycle, easy to raise on a large scale, low cost of feeding, and high production of eggs. Moreover, no co-infection by virus and bacteria were found in human and fish [9]. However, fish as bioreactors remain to be extensively studied. Fish eggs were first tested as potential bioreactors to express foreign recombinant proteins [10]. Human pharmaceutical proteins can be produced from fertilized eggs since human seventh coagulation factor can be widely expressed in tilapia embryos [11]. Recombinant goldfish luteinizing hormone can be successfully expressed and glycosylated in transgenic rainbow trout embryos [12]. Under the control of oocyte-specific gene *zp3* promoter, mature tilapia insulin-like growth factor was successfully expressed in zebrafish [9].

Many fish species, such as loach (*Paramisgurnus dabryanus*), yellow catfish (*Pelteobagrus fulvidraco*), and southern catfish (*Silurus meridoualis*), are able to release a large amount of mucus especially under stress conditions. Fish mucous cells are the most common cell types in the epidermis except for microfilament cells. Mucous cells have more endoplasmic reticulum structures and have mucus-secreting properties [13]. Fish mucus is known to function in breathing, ion and osmotic pressure regulation, reproduction, excretion, disease resistance, communication, feeding, and nesting [14,15,16]. Fish mucus is mainly composed of glycoproteins and contains other biologically active substances such as mucopolysaccharide [17], lysozyme [18], immunoglobulin [19], complement, carbonic anhydrase [20], lectin [21], secreted toxin [22], calmodulin [23], c-reactive protein [19], pheromones [24], and proteolytic enzymes [25]. Moreover, biologically active substances expressed in mucous cells are usually packaged in mucous bubbles and only when mucous cells migrate to the body surface can mucous substances be released [26], which would cause less harmful effects of foreign molecules on the donor body. Therefore, mucous cells in fish skin have the potential to produce biologically active molecules through collection of mucus.

To generate mucus gland bioreactors, the first step is to obtain a mucus cell-specific promoter to specifically control the expression of DNA sequences encoding a protein or peptide of interest and allow the secretion of the products into mucus. Keratin is a major class of intermediate filament proteins that are mainly expressed in the epidermis of higher eukaryotes. Three keratin genes have been identified to express in the epidermis of the skin [27,28], in which the *krt8* gene has been used as a zebrafish ideal epidermal marker during development. The promoter of the *krt8* gene (2.2 k) was used to specifically drive the expression of GFP reporter gene in the epidermis of the skin, both in zebrafish and barley [29,30]. Thus, the *krt8* gene promoter appears to be useful for generation of a mucus bioreactor.

The second step is to select a suitable DNA sequence encoding an important protein or peptide, whose biological activities can be easily detected either in cultured cells or animals. Grass carp hemorrhagic disease is a disease induced by grass carp reovirus (GCRV). The disease caused by GCRV leads to a mortality rate of more than 80% for young grass carp [31] and a severe loss to aquaculture every year in Asia. GCRV is a double-stranded RNA virus composed of 11 RNA subunits [32]. GCRV873 belongs to the type I GCRV and its genome sequencing has been completed [33]. GCRV873 can cause pathological changes of kidney tissue cell lines of grass carp (CIK) [34], suggesting CIK cells are suitable for in vitro assays of GCRV873 infection. The virus begins to replicate after CIK cells are infected with 12 h at 28 °C, and the virus begins to multiply in large numbers after 24–72 h. In addition, GCRV can infect rare minnow (*Gobiocypris rarus*) and black carp (*Mylopharyngodon piceus*), leading to damaged tissues with phenotypes similar to those of grass carp hemorrhagic disease [35,36].

Interferons (IFNs) are a class of cytokines with antiviral, anti-proliferative and immunomodulatory activities. These properties of IFNs are mainly used for treatment of virus diseases in human and animals. Type I interferon (IFNα/β) can be produced in large quantities by most cell types in response to viral or other microbial infections and plays an important role in immune response against multiple viruses [37]. Type I interferon can activate the JAK-STAT signaling pathway after binding to IFNAR1 and IFNAR2, followed by induction of downstream gene expression and exertion of their biological functions [38]. Thus, GCRV873 and IFNs system can be used to test the development of novel bioreactors.

In this study, we successfully generated a transgenic loach line expressing the type I grass carp IFN (gcIFN1) in the mucus. We tested the antiviral capability of gcIFN1 from cultured cells and mucus of transgenic loach. Our data indicate that fish mucus cells have a great potential for the generation of novel bioreactors.

## 2. Results

### 2.1. Construction of a Transgenic Vector

**Figure 1:**
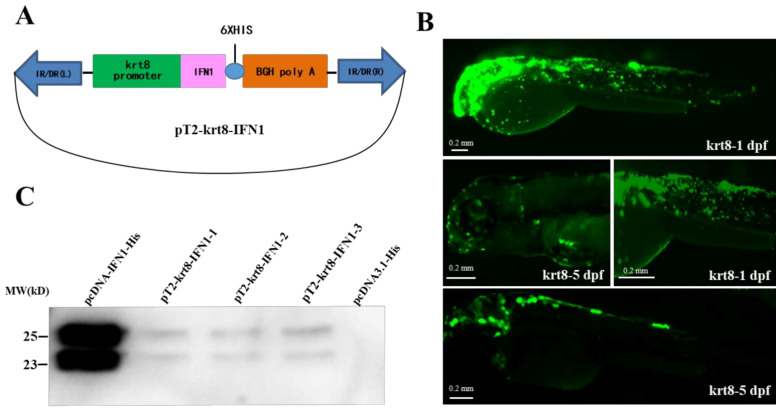
Construction of a transgenic vector. (**A**) Plasmid map of the transgenic vector containing a *krt8* promoter, grass carp interferon (IFN1) cDNA and His-tag. (**B**) The expression of EGFP under fluorescence microscope. krt8-1dpf: One day after fertilization; krt8-5dpf: five days after fertilization. (**C**) Western blot analysis of IFN1 in 293T cells. Group pcDNA-IFN1-His: 293T cells were transfected with pcDNA-IFN1-His; pT2-krt8-IFN1-1, 2 and 3: 293T cells were transfected with pT2-krt8-IFN1; Group pcDNA3.1-His: 293T cells were transfected with pCDNA3.1-His.

We have generated a transgenic vector pT2-krt8-IFN1, which contains the *krt8* promoter, the grass carp IFN1 cDNA, His-tag sequence at the 3′-end of IFN1 and a poly (A) signal (Figure 1A). To detect the activity of the *krt8* promoter, the IFN1 in pT2-krt8-IFN1 was replaced with EGFP to generate a testing vector pT2-krt8-EGFP. After microinjection of pT2-krt8-EGFP into fertilized eggs of loach, a large amount of green fluorescence signals were observed on the skin of larvae at 1–5 dpf (day post-fertilization) (Figure 1B). These data indicate that the *krt8* promoter can specifically drive the expression of EGFP in the skin of transgenic loaches.

After transfection of 293T cells with pcDNA3.1 or pcDNA-IFN1-His, the supernatant medium was collected for detection of IFN1 (Supplementary Figure S1A). Western blotting showed that IFN1 can be secreted to the medium of cultured 293T cells (Supplementary Figure S1B). Moreover, transfection of 293T cells with the transgenic vector pT2-krt8-IFN1, a small amount of IFN1 can be detected in the culture medium (Figure 1C), indicating that IFN1 expressed under the control of *krt8* promoter can be secreted outside the cells.

### 2.2. Antiviral Activity of Grass Carp IFN1

To further test the antiviral activity of grass carp IFN1, we performed plaque formation assays. We found that the titer of the virus was 5.0 × 10^7^ PFU/mL (Figure S2A). When the dilution factor of GCRV873 reached to 10^6^ times, CIK cells showed an obviously pathological phenomenon of forming plaques. Thus, the appearance of plaques can be a marker for detecting the antiviral activity of proteins expressed in vitro.

**Figure 2:**
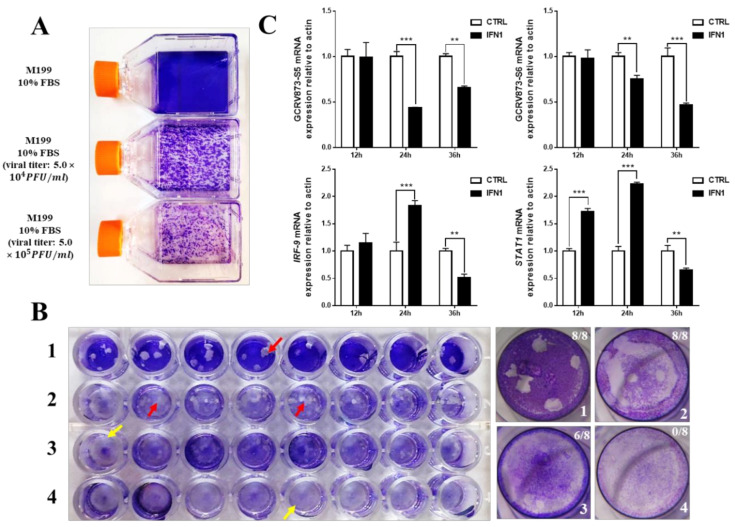
Antiviral ability of grass carp IFN1. (**A**) The pathogenicity of GCRV873 virus particles. The upper bottle contains M199 culture medium supplied with 10% fetal bovine serum; the middle bottle contains M199 culture medium supplied with 10% fetal bovine serum and GCRV873 virus particles at a final concentration of 5.0 × 10^4^ PFU/mL; the lower bottle contains M199 culture medium supplied with 10% fetal bovine serum and GCRV873 virus particles at a final concentration of 5.0 × 10^5^ PFU/mL. (**B**) The results of crystal violet staining of CIK cells in M199 medium supplied with or without GCRV873 virus particles. 1: CIK cells were infected with GCRV873 (viral titer: 1.0 × 10^2^ PFU/mL); 2: CIK cells were electrically transfected with pCDNA3.1 and infected with GCRV873 (viral titer: 1.0 × 10^2^ PFU/mL); 3: CIK cells were electrically transfected with pCDNA-IFN1-His and infected with GCRV873 (viral titer: 1.0 × 10^2^ PFU/mL); 4: CIK cells were electrically transfected with pCDNA-IFN1-His and not infected with GCRV873. Red arrow: Plaques formed in cultured CIK cells. Yellow arrow: No plaques formed in cultured CIK cells. (**C**) The transcriptional expression of factors in CIK cells infected with GCRV873 (viral titer: 1.0 × 10^2^ PFU/mL). S5 and S6 are subunits of GCRV873. IFR-9 and STAT1 are key molecules downstream of type I interferon signaling. Data were expressed as mean ± SD. ** *p* < 0.01; *** *p* < 0.001.

To determine the antiviral activity of IFN1 expressed in 293T cells, antivirus tests were carried out using CIK cells and GCRV873 virus particles. When the density of CIK cells reached to about 80% confluence, culture medium containing different concentrations of GCRV873 virus particles was added. As shown in Figure 2A, CIK cells that were subjected to infection with GCRV873 virus particles (Viral titer = 5.0 × 10^4^ PFU/mL or 5.0 × 10^5^ PFU/mL) underwent severe apoptosis, indicating that RNA virus particles of GCRV873 could actively infect CIK cells and caused apoptosis.

Next, pcDNA3.1 or pcDNA-IFN1-His plasmids were electrically transfected into CIK cells at about 80% confluence. A final concentration (Viral titer = 1 × 10^2^ PFU/mL) of GCRV873 in M199 medium containing 2% fetal bovine serum was used for different experimental groups after transfection for 12 h. We found that cells expressing IFN1 formed less number of plaques than the control cells transfected with pcDNA3.1 (Figure 2B), suggesting an antiviral activity of grass carp IFN1 from CIK cells.

To further detect the antiviral activity of grass carp IFN1 from CIK cells, total RNAs of cultured CIK cells at different time points of transfection (Supplementary Figure S2B) were extracted to detect the transcription of S5 and S6 subunits of GCRV873 as well as STAT1 and IFR-9 that are regulated by IFN1 signaling. In comparison with those in control cells transfected with pcDNA3.1 plasmids, mRNA levels of S5 and S6 subunits were significantly down-regulated in cells transfected with pcDNA-IFN1-His plasmids for 24 and/or 36 h. However, mRNA levels of STAT1 and IFR-9 in cells transfected with pcDNA-IFN1-His plasmids were significantly induced from 12 to 24 h and decreased at 36 h (Figure 2C). These data indicate that cell-expressed grass carp IFN1 can inhibit the replication of the GCRV873 and activate the IFN signaling pathway.

### 2.3. Generation of Transgenic Loaches

**Figure 3:**
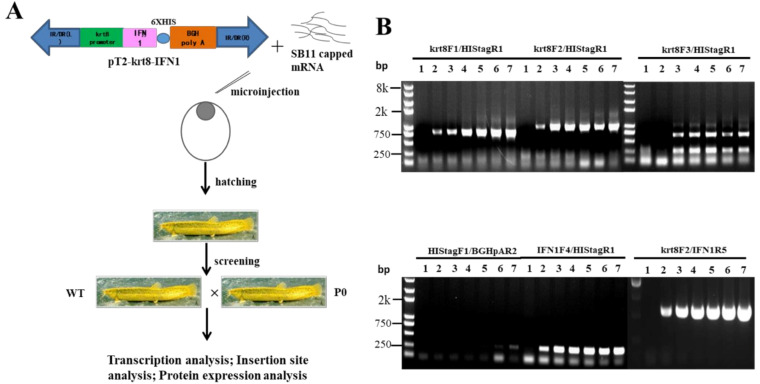
Generation of transgenic loach. (**A**) The strategy of generation of transgenic loach. (**B**) The sensitivity and specificity of seven primer pairs were determined in a 25 μL volume containing 100 ng genomic DNA and transgenic plasmids pT2-krt8-IFN1 (0, 1, 5, 10, 20, 50 or 100 copies) as template.

A strategy for SB transposon-mediated generation of transgenic loaches was presented in Figure 3A. Briefly, the transgenic plasmids pT2-krt8-IFN1 were microinjected with capped SB11 mRNA into one-cell stage fertilized eggs. Injected eggs were hatched out and reared to adult for transgenic screening. Positive founder loaches (P0) were individually crossed with wild type (WT) loaches to produce F1 offspring.

Seven PCR primers (Table 1) were designed on the *krt8* promoter and IFN1-coding sequence of grass carp. The transgenic plasmids were diluted into different copy numbers as templates to optimize primer pairs for the screening of transgenic loaches.

As shown in Figure 3B, two primer pairs, krt8-F1/Histag-R1 and krt8-F2/IFN1-R5, exhibited better screening results than others. Thus, all of positive loaches in the study were screened with these two primer pairs, krt8-F1/Histag-R1 and krt8-F2/IFN1-R5.

Some of the electrophoresis gel diagrams for PCR are shown in Supplementary Figure S3 and positive rates of P0, F1, and F2 generations were 3.46%, 6.62%, and 24.2%, respectively (Table 2). These results indicate that we have successfully obtained a transgenic line in which IFN1 cDNA can stably transmit to the following offspring.

### 2.4. Expression of Grass Carp IFN1 in Transgenic Loaches

**Figure 4:**
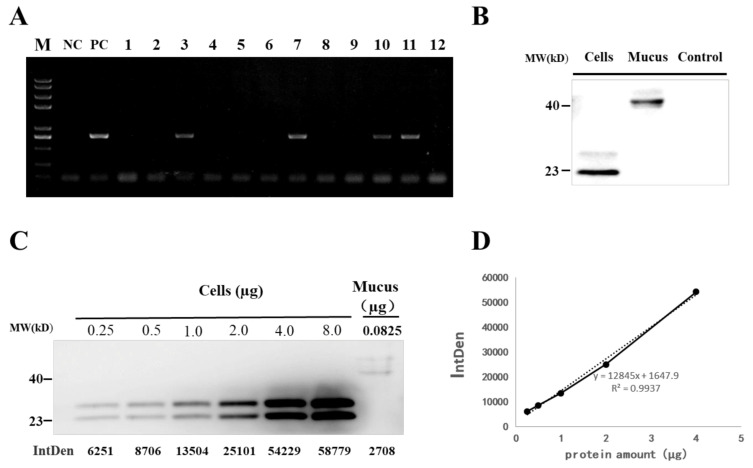
Detection of grass carp IFN1 in transgenic loach. (**A**) Detection of the IFN1-expressing cassette in F1 offspring by PCR. NC: negative control; PC: positive control; 1–12: F1 loaches. (**B**) Detection of grass carp IFN1 in mucus using western blots. Cells: the supernatant medium of cultured 293T cells transfected with pcDNA-IFN1-His; Mucus: the mucus collected from F1 loaches containing IFN1-expressing cassette; Control: the mucus collected from wide type loaches. (**C**) Quantitative analysis of grass carp IFN1 in mucus using western blots. Cells: Purified protein from the supernatant medium of 293T cells transfected with pcDNA-IFN1-His; Mucus: the mucus collected from F1 loaches containing IFN1-expressing cassette. (**D**) The linear analysis of gray values (IntDen) and protein concentrations in the supernatant medium of 293T cells transfected with pcDNA-IFN1-His to determine the concentration of grass carp IFN1 in the mucus collected from F1 loaches containing IFN1-expressing cassette in (**C**).

To address whether the integration of IFN1 cDNA-containing cassette in the genome of transgenic loaches can properly secrete grass carp IFN1 into mucus, the genome DNA from tail fins of F1 loaches was isolated for detection of the IFN1 cDNA with a primer pair of krt8-F1/Histag-R1. As shown in Figure 4A, 4 out of 12 F1 individuals were positive transgenic loaches showing DNA bands with the same size as that of the positive control (PC) and confirmed by DNA sequencing.

Next, we detected the existence of grass carp IFN1 in mucus of positive loaches. In comparison with the scraping approach, collection of the mucus through over-extrusion gave less cell debris (Supplementary Figure S4A). After centrifugation and filtration, mucus samples from positive and wild type loaches were subjected to western blotting with His-tag antibody. Two clear protein bands appeared in the mucus of positive loaches but not in wild type loaches. However, the molecule weight of IFN1 in the mucus was higher than that of IFN1 from cultured 293T cells (Figure 4B). Moreover, purified grass carp IFN1 from 293T cells (Supplementary Figure S4B) was found to be modified by *N*-glycosylation (Figure S4C), but IFN1 from mucus could not be deglycosylated with PNGase F or *O*-glycosidase (data not shown). These date suggest that integrated IFN1-cDNA can be translationally expressed and expressed grass carp IFN1 in mucus of transgenic loaches appear to be not modified by *N*- and *O*-glycosylation.

To determine the efficiency of mucus gland bioreactor, we further measured the content of IFN1 in the mucus of transgenic loaches. IFN1 expressed in cultured 293T cells was purified and dyed with coomassie bright blue on SDS-PAGE (Supplementary Figure S4B). The purified IFN1 can be used as a reference protein to detect the content of IFN1 in mucus. Different amounts of purified IFN1 and 20 µL mucus of transgenic loaches were examined with western blotting (Figure 4C) and approximately 0.0825 µg of IFN1 in 20 µL mucus of transgenic loaches was determined by analysis with the Image J software (Figure 4D). Thus, loach mucus can be used to generate bioreactors for the production of proteins or peptides.

### 2.5. IFN1 in Mucus of Transgenic Loaches Protects CIK Cells from Viral Infection

**Figure 5:**
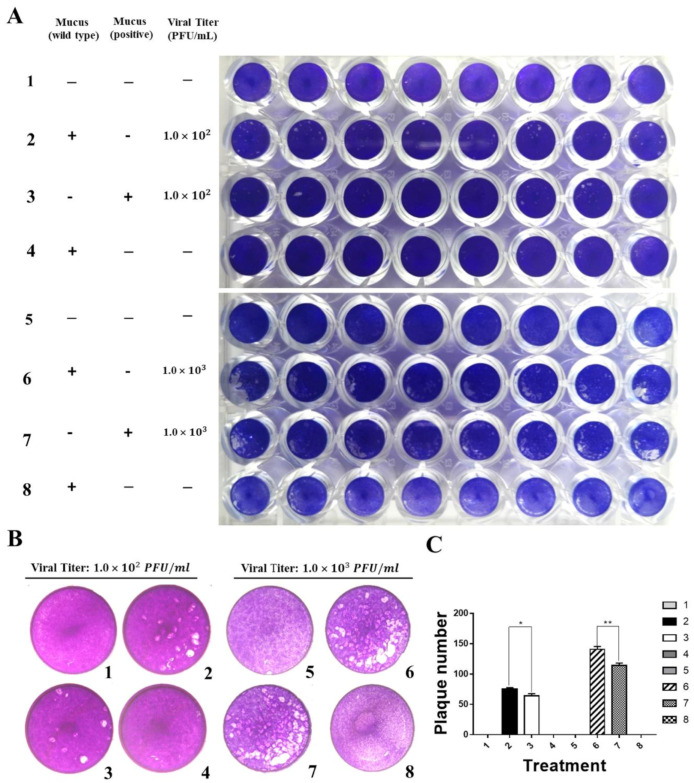
Grass carp IFN1 from mucus inhibited the plaque formation of cultured cells infected with GCRV873. (**A**,**B**) Detection of the antiviral ability of grass carp IFN1 from the mucus of transgenic loach. 1, 5: added M199 medium containing 2% FBS; 2: added M199 medium containing 2% FBS and 20% mucus (*v*/*v*) from wide type (WT) loaches and infected with GCRV873 (viral titer: 1.0 × 10^2^ PFU/mL); 3: added M199 medium containing 2% FBS and 20% mucus (*v*/*v*) from grass carp IFN1-expressing transgenic loaches and infected with GCRV873 (viral titer: 1.0 × 10^2^ PFU/mL); 6: added M199 medium containing 2% FBS and 20% mucus (*v*/*v*) from WT loaches and infected with GCRV873 (viral titer: 1.0 × 10^2^ PFU/mL); 7: added M199 medium containing 2% FBS and 20% mucus (*v*/*v*) from grass carp IFN1-expressing transgenic loaches and infected with GCRV873 (viral titer: 1.0 × 10^3^ PFU/mL); 4, 8: added M199 medium containing 2% FBS and 20% mucus (*v*/*v*) from WT loaches. (**C**) Graph of plaque numbers of the Figure 5A. The experiments were performed in triplicate. Data were expressed as mean ± SD. * *p* < 0.05; ** *p* < 0.01.

To determine whether IFN1 in mucus of transgenic loaches has an antiviral activity, the ability of CIK cells to form plaques was detected after infection with GCRV873. CIK cells were cultured in 2% M199 medium containing 20% volume of mucus from transgenic loaches. Then, a proper viral titer of GCRV873 which was 1.0 × 10^2^ PFU/mL or 1.0 × 10^3^ PFU/mL was added in medium. The mucus from wide-type loaches was used as the control. Assays were terminated by addition of 4% PFA fixing and a crystal violet staining solution, allowing visualization of the plaques (Figure 5A,B).

At a viral titer of 1 × 10^2^ PFU/mL, plaque numbers on plates containing IFN1 reduced by 16% when compared to those of the control. Similarly, at a viral titer of 1 × 10^3^ PFU/mL, plaque numbers on plates containing mucus from transgenic loaches reduced by 18% in comparison with those of the control (Figure 5C). These results indicate that mucus of IFN1-transgenic loaches can confer a protective effect on CIK cells against GCRV873 infection.

### 2.6. Insertion Site of IFN1 cDNA in Transgenic Loaches

**Figure 6:**
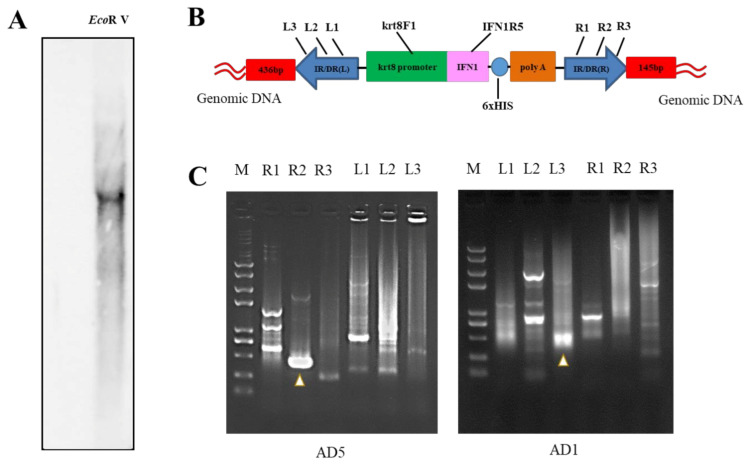
Analysis of integration site of IFN1-expressing cassette in transgenic loaches. (**A**) Genomic DNA of grass carp IFN1 cDNA-transgenic loaches was digested with *Eco*RV and subjected to Southern blotting with digoxigenin-labelled cDNA probes and antidigoxin antibodies. (**B**) Schematic diagram of the transposon-mediated IFN1-expressing cassette integrated in the genome, showing primer pairs krt8-F1/IFN1-R5 for Southern blots. (**C**) The electrophoresis of genome walking. AD5 and AD1 are merging primers for the genome of loach. R1, R2, R3 and L1, L2, L3 are primers designed on integrated IFN1-expressing cassette of the plasmid pT2-krt8-IFN1.

Although the stable transmission and expression of IFN1 cDNA were determined in transgenic loaches, the copy numbers and integration sites of IFN1-expressing cassette in the genome of transgenic loaches need to be characterized. After digestion of the genomic DNA with *Eco*RV, a single copy of IFN1 cDNA was detected by Southern blots to stably integrate into the genome of transgenic loaches (Figure 6A) and two primers krt8F1 and IFN1R5 used for amplification of DNA probes were shown in Figure 6B.

Next, genome walking assays were performed to determine the integration site of IFN1-expressing cassette in transgenic loaches. We designed the primer pairs L1, L2, L3 and R1, R2, R3 on the IFN1-expressing cassette (Table 1 and Figure 6B). The left or right junction sequences at the insertion site of IFN1-expressing cassette in the transgenic loach was further determined by PCR amplification. Figure 6C demonstrated the electrophoretic gel maps for PCR using degenerate primers of AD5 and AD1. DNA sequencing indicated that the 436-bp and the 145-bp DNA fragments contained loach genome sequences adjacent to the left and right terminus of IFN1-expressing cassette, respectively (Figure S5). Although the precise location of the inserted cassette within the loach genome remain to be determined due to the lack of loach genomic data, these data suggest that we have successfully generated a transgenic loach line, in which a single copy of the IFN1-expressing cassette has stably integrated into the loach genome.

## 3. Discussion

Bioreactors developed in bacteria, yeast, plants and animals always exhibit some defects in expression, activity or purification of biologically active molecules [39], so the development of novel and effective bioreactors remains a high priority task for biopharmaceutical industry. In this study, we have successfully generated a transgenic loach line in which a single copy of grass carp IFN1-expressing cassette has stably integrated into the loach genome. Purified IFN1 from cultured 293T cells remained a strong antiviral ability. Moreover, the proper expression of IFN1 in mucus of transgenic loaches was detected by Western blotting. Hence, mucus glands of transgenic fish including loach exhibited the potential to become a novel bioreactor for the production of protein/peptides with medical and industrial values.

The development of highly effective bioreactors has always been a hot research topic due to an increasing market demand for protein/peptide drugs [40]. As of May 2017, 74 antibody-based molecules have been approved by a regulatory authority in a major market [41]. In addition to advantages, bioreactors currently available have demonstrated various disadvantages. Bacteria bioreactors have a poor capacity to synthesize some proteins and particularly those containing a complex structure [42]. Yeast bioreactors can synthesize and secrete a human glycoprotein with uniform complex *N*-glycosylation, which could be used for elucidating the structure-function relation of glycoproteins [43]. However, recombinant hepatitis B vaccine prepared from yeast does not contain the disulphide bridges that must be synthesized [44]. Transgenic plants producing antibodies may raise environmental concerns, whereas such problems are unlikely to be encountered with transgenic animals that are kept in enclosed areas [45]. Animal mammary gland bioreactor such as saliva [46] and other transgenic product such as blood [47] have proved to be a production platform for foreign proteins. Recombinant protein produced by transgenic animals is a cumbersome process and remains problematic in the application of this technology due to high cost and low expression efficiency [48]. Our data in this study indicate that the bioreactor of fish mucous gland exhibited the potential to overcome some of these defects, such as secreting a large amount of mucus under stimulation without harmful effects on itself and relatively low cost for the isolation of recombinant proteins/peptides from mucus.

In the prevention and treatment of grass carp haemorrhagic disease, intraperitoneal injection and drug immersion are two main therapeutic approaches. Induced interferon from crucian carp at 10,000 U/mL shown antiviral activity: the dose of intraperitoneal injection was 0.3 mL/trail and the concentration of drug immersion was 50 U/mL [49]. The mucus from positive loaches has showed a modest antiviral ability due to the low content of expressed INF1, so further efforts are needed to improve the secretion of proteins/peptides by fish mucous glands.

The *krt8* promoter used in this study can drive the expression of genes in the skin of both zebrafish and medaka [29,30]. A controlled and localized expression of genes in fibroblasts can be improved under the control of collagen-specific promoter [50]. Thus, the promoter used in this study could be replaced with a mucus gland-specific promoter to increase the specific expression and secretion of target proteins/peptides.

Difference in integration sites could affect the expression level of foreign genes within a target genome. Transposon systems such as the Sleeping Beauty transposon [51] and the Tol2 transposon system [52] can significantly improve the integration efficiency of foreign genes. Transgenic bioreactors containing limited copies of foreign genes that have integrated into non-coding regions of the target genome could increase the expression of target proteins and reduce side-effects on the development and growth of transgenic animals. In addition, many fish species such as Yellow River catfish have more mucous cells and a stronger ability to secrete mucus [34] could be used for development of mucus gland bioreactors.

Mucus contains a large number of lectins and other substances [26]. During the process of secretion, target proteins may covalently bind to other substances, thus affecting the activity of the protein. In this study, we found that *N*-glycosylation occurred in grass carp IFN1 secreted by in vitro cultured 293T cells, but not in grass carp IFN1 from mucus of transgenic loaches. It is likely that complicated modifications have occurred in grass carp IFN1 from mucus of transgenic loaches as evidenced by its increased molecular weight. Further investigations are required to purify a large amount of IFN1 from mucus of transgenic loaches and then determine the structure and modification forms. Identification of modification types and the residue within target proteins could improve the efficiency of loach mucus bioreactors. Moreover, molecular mechanisms of mucus secretion remain largely unclear. We have previously identified signal pathways related to mucus secretion through RNA-seq analysis of loach skin transcripts [53]. To further investigate the intracellular mechanisms of mucus secretion and testing approaches such as low-voltage electricity and low-concentration salt to safely stimulate the secretion of IFN1 by mucus cells of transgenic fish will contribute to the development of mucous gland bioreactors in fish.

Taken together, we successfully produced transgenic loach that have the capability to stably express and secrete IFN1 in the mucus. IFN1 from transgenic loach remained the antiviral activity against GCRV replication and activated downstream genes of JAK-STAT signaling pathway. Fish mucus glands exhibited a great potential for production of proteins/peptides.

## 4. Materials and Methods

### 4.1. Maintenance of Loaches

Large scale loaches (average body weight 25.28 ± 4.55 g) were purchased from a local fish market in Wuhan, Hubei Province of China, and reared in a recirculating water system at 28 °C with commercial feeds twice a day.

### 4.2. Generation of Transgenic Loaches

A DNA fragment encoding the type I interferon (IFN1, AY452069.1) of grass carp was subcloned into the pT2-HB vector (obtained from Perry Hackett’s laboratory, St. Paul, MN, USA) to generate a transgenic vector pT2-krt8-IFN1. A *krt8* promoter was obtained from zebrafish genome using the primer pair krt8-F/krt8-R (Table 1) and inserted at *Hin*dⅢ/*Eco*RⅠ sites upstream of the IFN1 cDNA that was tagged with a histidine chain at the 3’-end of IFN1. Total RNAs for cDNA synthesis and detection of transgene expression were extracted with the TRIzol reagent (15596026, Invitrogen, Carlsbad, CA, USA) and a Revert Aid first-strand cDNA synthesis kit (27926101, GE, Boston, MA, USA). The sequences of DNAs or proteins were blasted to the NCBI database and all PCR primers were designed with Vector NTI.

The vector pSB11RNAX was obtained from Perry Hackett’s laboratory at university of Minnesota and linearized to generate capped SB11 mRNA in vitro using the mMESSAGE kit from Ambion (Austin, TX, USA) according to the manufacturer’s instructions.

Mature and healthy male and female loaches were maintained at two different water tanks until artificial breeding as previously described [54]. Fertilized eggs were checked under a stereomicroscope by the blastodisc formation and eggs showing a fertilization rate of more than 80% were used for microinjection. One-cell stage embryos were injected with 100 ng/µL capped SB11 mRNA and 25 ng/µL linearized pT2-krt8-EGFP/pT2-krt8-IFN1. Microinjected embryos were cultured in aeration water at 28 °C for subsequent experiments. Aerated water was changed once a day and dead embryos were removed. Fairy shrimp and egg yolk were used as the opening food of loach larvae.

### 4.3. Cell Culture, Transfection and IFN1 Purification

Human embryonic kidney 293 cells (293T) were cultured in DEME culture medium (SH30022.01B, Hyclone) supplemented with 10% fetal bovine serum. Kidney tissue cell lines of grass carp (CIK) were cultured in M199 culture medium (SH30253.01B, Hyclone, Logan, UT, USA) supplemented with 10% fetal bovine serum (SH30084.02, Hyclone, Logan, UT, USA). 293T cells are widely used for in vitro expression of proteins due to its rapid growth and strong secretion ability. CIK cells were used in antiviral experiments since GCRV873 infection can cause severe cytopathic effects such as the formation of cell plaques.

The pcDNA-IFN1-His was constructed by subcloning the IFN1 cDNA into the pcDNA3.1 vector for in vitro synthesis of IFN1 in cultured 293T cells. When the density of 293T cells reached to above 80% confluence, the medium was replaced with serum-free medium. The expression vector pcDNA-IFN1-His was then transfected into 293T cells with the Lipofectamine 2000 Reagent (11668019, Invitrogen, Carlsbad, CA, USA) or using electric transfection (Biorad, Hercules, CA, USA). After 12 h of transfection, the medium was replaced with 10% fetal bovine serum. To prevent the interference of proteins in medium, serum-free medium was replaced after 24 h. About 200 µL supernatant media was daily collected for IFN1 purification.

After filtration and centrifugation to remove the impurities in the supernatants, the solution containing IFN1 was purified with nickel ion columns, which showed a strong affinity for the His-tag at the 3′-end of IFN1. Different buffers used for the purification process include lysis buffer (250 mM Tris-HCl, pH 8.0; 150 mM NaCl), wash buffer (250 mM Tris-HCl, pH 8.0; 150 mM NaCl; 15 mM imidazole) and elution buffer (250 mM Tris-HCl, pH 8.0; 500 mM imidazole). Protein solutions during the purification process were collected into multiple tubes and loaded on SDS-PAGE gels followed by coomassie brilliant blue staining to determine the protein quality.

### 4.4. Antiviral Assays

To detect the antiviral activity of IFN1, the expression vector pcDNA-IFN1-His was transfected into CIK cells and the medium was replaced with medium containing virus particles of GCRV873 after 12 h. The virus begins to replicate after CIK cells are infected for 12 h at 28 °C, and multiply in large numbers after infection for 24–72 h [36,37]. Hence, we collected the sample at 12 h, 24 h, and 36 h after GCRV873 infection. PCR primers were designed to detect the mRNA expression of target genes. S5 and S6 are the subunits of GCRV873. STAT1 and IRF-9 are two key genes downstream of JAK-STAT signaling pathway, which can be activated by IFN1 binding to IFNAR1 and IFNAR2 [38]. The number of plaques formed in different cell dishes were detected with crystal violet staining to determine the antiviral ability of IFN1. The virus of GCRV873 was from Qin Fang’s laboratory at Wuhan Institute of Virology, Chinese Academy of Sciences.

PCR primers for detecting the S5 and S6 subunits of GCRV873 and the factors downstream of IFN1 signaling such as STAT1 and IFR-9 were shown in Table 1.

### 4.5. RT-PCR

Five primer sets were designed according to the sequence of pT2-krt8-IFN1 (Table 1) and RT-PCR assays were conducted to detect the transcription of IFN1 gene. Total RNA was extracted from tail fin and treated with RNase-free DNase I to eliminate contaminated DNA before reverse transcription. Wild type loach was used as a negative control, and the transgenic plasmid was used as a positive control. The PCR procedure was: 94 °C for 5 min; 30 cycles at 94 °C for 30 s, 55 °C for 30 s, and 72 °C for 40 s; finally, 72 °C for 10 min.

### 4.6. Western Blotting

Mucus was collected through either the over-extrusion (putting 4 or more loaches into a collection bag) or scraping with a spoon. The resulting mucus was treated with a 0.22 µm filter to remove impurities such as cell debris and then centrifuged at 12,000 rpm at 4 °C. The supernatants were collected and quantified using BCA Protein Assay Kit (P0011, Beyotime, Shanghai, China). Western blotting assays were performed following our previous protocol [55]. Briefly, western blotting membranes were subjected to primary antibodies against His-tag (71841, Novagen, Darmstadt, Germany) and then probed with HRP-conjugated secondary antibody (BA1050, Boster, Wuhan, China). Recombinant IFN1 from cultured 293T cells was used as the positive control.

### 4.7. Deglycosylation of Purified Protein

The concentration of purified proteins was measured using UV-Vis Spectrophotometer (Quawell Q5000, Sunnyvale, CA, USA). Detection of *N*-deglycosylation, *O*-deglycosylation, *N*- and *O*-deglycosylation: the reaction was performed in a total volume of 10 µL containing 20 µg purified protein, 1 µL of 10 × glycoprotein denaturing buffer and ultrapure water. The reaction mixture was heated in boiling water for 10 min, cooled to room temperature (25 °C), added with 2 µL of 10 × glycobuffer2, 2 µL of 10% NP-40, 1 μL of PNGase F (P0704S, NEB, Ipswich, MA, USA) or 1 μL of *O*-glycosidase (P0733S, NEB, Ipswich, MA, USA) or 0.6 μL of PNGase F and 0.6 μL of *O*-glycosidase, and ultrapure water in a total volume 20 μL, and incubated at 37 °C for 2 h.

### 4.8. Southern Blotting

Genomic DNA was extracted from the tail fin of F1 transgenic loaches. 5 µg of genomic DNA was digested with *Eco*RV at 37 °C overnight. Digested DNA was separated on 0.7% agarose gel and then transferred to nylon membranes (RPN303B, Amersham, Shanghai, China) using Semi-dry electrometer (1703940, Biorad, Hercules, CA, USA). The reagents and conditions for the membrane transfer were referred to the instructions of the semi-dry film transfer apparatus. The probes were amplified from pT2-krt8-IFN1 coding sequence with primers (krt8-F1: CAGAGGGACTTTGACTCTCCTTTG; IFN1-R5: CGTCCTGGAAATGACACCTTGG) (Table 1) and labeled with the DIG High Prime DNA Labeling and Detection Starter Kit II from Roche. Hybridization and immunological detection were processed according to the manufacturer’s procedures.

### 4.9. Genome Walking Assays

Genomic DNA was extracted from F1 transgenic loaches. Genome walking assays were performed according to the Genome Walking Kit protocol from TaKaRa (Dalian, China) with some modifications to obtain chromosomal DNA sequences flanking the integration sites of SB transposons. AD1 and AD5 were designed as degenerate primers according to the design method of degenerate primers [56,57]. IR/DR(L)-specific primers were L1, L2 and L3. IR/DR(R)-specific primers were R1, R2 and R3. Details of all primers were shown in Table 1.

PCR products were detected by loading on 1.5% agarose gel. A specific DNA fragment in the second/third round PCR products was 100-bp smaller than the corresponding products from the first/second round PCR, which was isolated and cloned into the pZero2-TA vector for sequencing.

### 4.10. Statistical Analysis

All the experimental data from at least three independent experiments were analyzed using GraphPad Prism 7.0 software (La Jolla, CA, USA) and expressed as the mean ± SD. Student’s t-test were performed to compare the differences between two groups.

## 5. Patents

We have filed Chinese and American patents entitled “Method for preparing fish skin mucous gland bioreactor and application thereof”. The Chinese patent number is 201910912452.X. The American patent is pending and the application claims priority under 35 U.S.C. 119(a–d) to CN 201910912452X, filed on 25 September 2019.

## Figures and Tables

**Table 1:** Primers used in this study.

Primer	Sequence (5′-3′)
gcIFN1-F	CTCGAGATGAAAACTCAAATGTGGACG
gcIFN1-R	CCTAGGAGCAGACAACCGTTACGAAC
Krt8-F1	CAGAGGGACTTTGACTCTCCTTTG
HIS tag-R1	ATGATGATGATGATGATGGTCG
Krt8- F2	GAATGCCTGTCCTCAAGTCTCAAG
IFN1-R5	CGTCCTGGAAATGACACCTTGG
IFN1-F2	CGATACAGGATGATAAGCAACGAG
IR-F1	CTGTATCACAATTCCAGTGGGTC
krt8-R5	GGCATTTAATAGCATTACGCAATCG
krt8-F	CCTTCCCTTCTAAGTCTGACG
krt8-R	GATGCCTGTGTCTTTGAGTTG
GCRV873-S5-F	GTGGCACGGCTCTGCAAGTT
GCRV873-S5-R	CAACCGAGGCACCATCAACCAT
GCRV873-S6-F	TGCGACAACGGCTGCTTTGAT
GCRV873-S6-R	TTGCGGACAACCAACGGATGG
STAT1-F	AGACCAGCAAGACGAATACGA
STAT1-R	TGTTGACGGCACCTCCATT
IRF-9-F	GCTGGACATCTCAGAACCTTAC
IRF-9-R	CTCCTCCTGCTGCTCCTTAC
IFN1-F2	CGATACAGGATGATAAGCAACGAG
krt8-R5	GGCATTTAATAGCATTACGCAATCG
AD1	TGWGNAGWANCASAGA
AD5	STAGNATSGNGTNCAA
R1	ATGTAAACTTCTGACCCACTGGGAATG
R2	TGGTGATCCTAACTGACCTAAGACAG
R3	CGACTTCAACTGAGTCGACCTCG
L1	TCAGACTTAGAAGGGAAGGAAGC
L2	AGTAGATGTCCTAACTGACTTGCC
L3	ATAGTGAGTCGTATTACGCGCGCT
IR-F1	CTGTATCACAATTCCAGTGGGTC
Actin-F	CGAGCAGGAGATGGGAACC
Actin-R	CAACGGAAACGCTCATTGC

**Table 2:** PCR results of transgenic loaches.

Generation	Total No. Detected	Positive No.	Positive Rates (%)
P0	260	9	3.46
F1	528	35	6.62
F2	95	23	24.2

## Data Availability

The data presented in this study are available in article and supplementary material.

## Supplementary Materials

The following are available online at https://www.mdpi.com/1422-0067/22/2/687/s1. Figure S1. Detection of grass carp IFN1 secreted from 293T cells. Figure S2. Determining the titer of GCRV873 virus and detecting the antiviral capability of recombinant IFN1. Figure S3. Detection of transgenic loaches containing IFN1-expressing cassette in P0 founders. Figure S4. The extraction of mucus from loaches and deglycosylation of purified grass carp IFN1 from 293T cells. Figure S5. The sequencing data of genome walking from transgenic loaches.

## Abbreviations

MDPI	Multidisciplinary Digital Publishing Institute
DOAJ	Directory of open access journals
PCR	Polymerase chain reaction
RT-PCR	Reverse transcription PCR
gcIFN1	Grass carp type I interferon
GCRV	Grass carp reovirus
CIK	Kidney tissue cell lines of grass carp
293T	Human embryonic kidney 293 cells
GFP	Green flourescent protein
PFU	Plaque forming unit
FBS	Fetal bovine serum
