# Supplementary material for: Development of a Mucus Gland Bioreactor in Loach Paramisgurnus dabryanus
Source: Int J Mol Sci. 2021 Jan 12;22(2):687. doi: 10.3390/ijms22020687 (PMC7827776; doi:10.3390/ijms22020687)
Supplement: Supplementary file 1 [file ijms-22-00687-s001.pdf]

SUPPLEMENTAL FIGURES AND LEGENDS

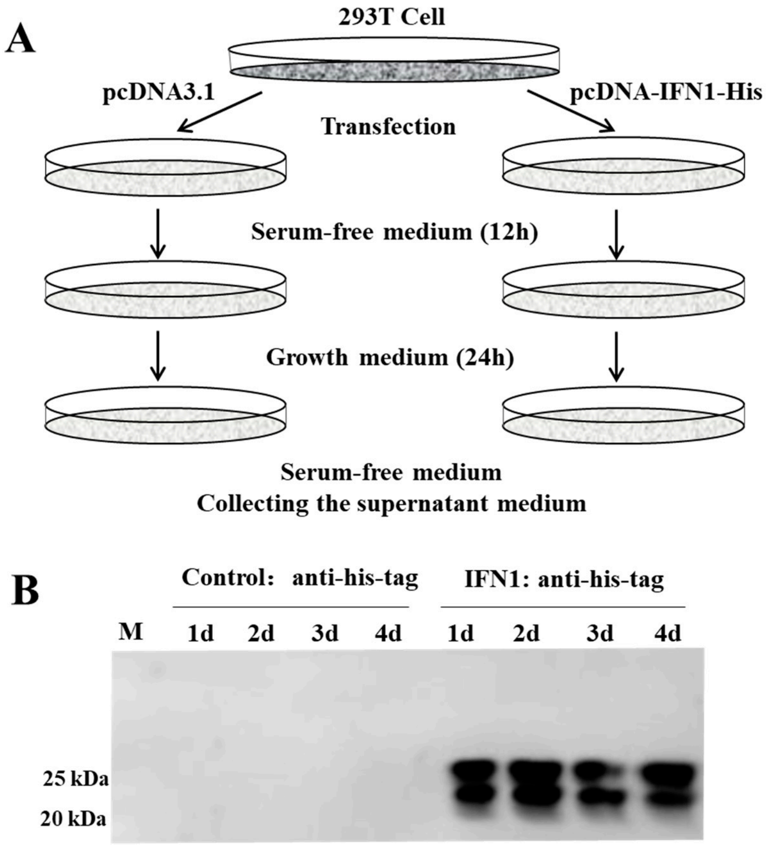

Figure S1. Detection of grass carp IFN1 secreted from 293T cells. (**A**)The steps of collecting recombinant interferon in vitro. 1:293T cells were transfected pcDNA3.1; 2: 293T cells were transfected with pcDNA-IFN1-His. (**B**)Western blot analysis of IFN1 in supernatant of 293T cells. The supernatant mediums were collected from the control (control: transfected with pCDNA3.1) and IFN1-expressing cells (IFN1: transfected with pCDNA-IFN1-His) at 1, 2, 3 and 4 days.

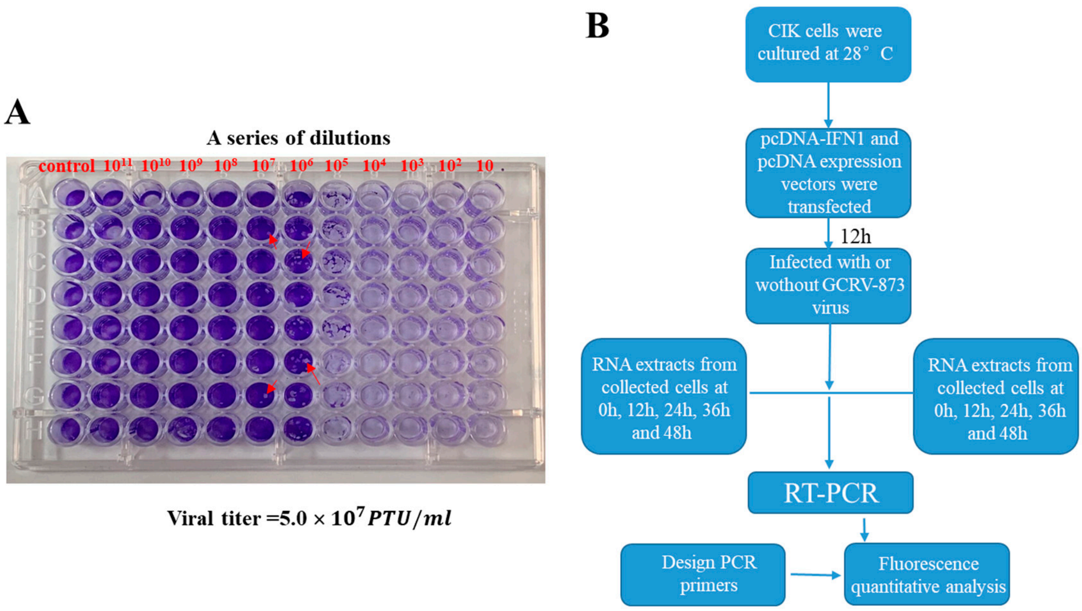

Figure S2. Determining the titer of GCRV873 virus and detecting the antiviral capability of recombinant IFN1. (A) Plaque assays of CIK cells infected with GCRV873 to determine the virus titer. Red arrow: CIK cells showing plaques. (B) The strategy for analyzing the antiviral ability of recombinant IFN1.

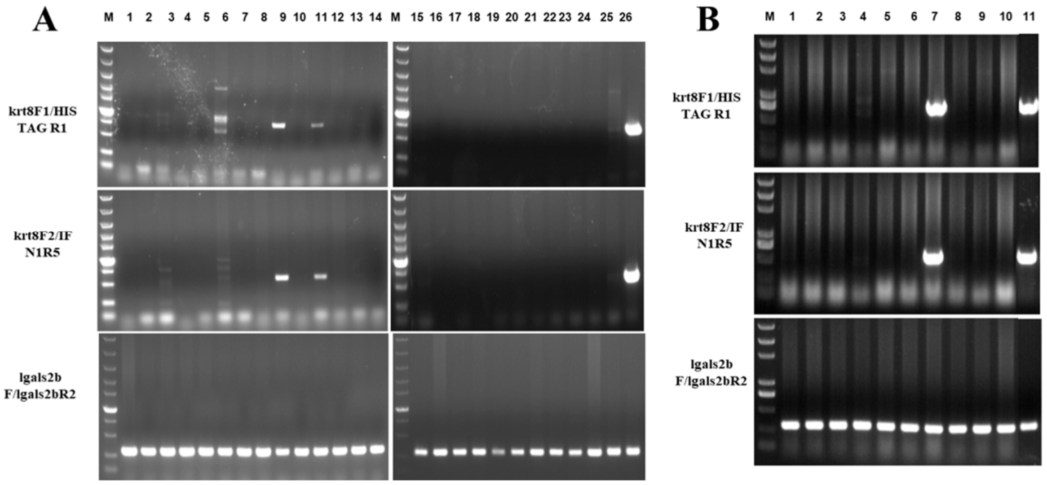

Figure S3. Detection of transgenic loaches containing IFN1-expressing cassette in P0 founders. Positive detection of P0 generation transgenic loaches. 1-24: genome DNA from P0 generation transgenic loaches; 25: negative control; 26: positive control; (B) Positive detection of F1 generation transgenic loaches; 1-9: genome DNA from F1 generation transgenic loaches; 10: negative control; 11: positive control.

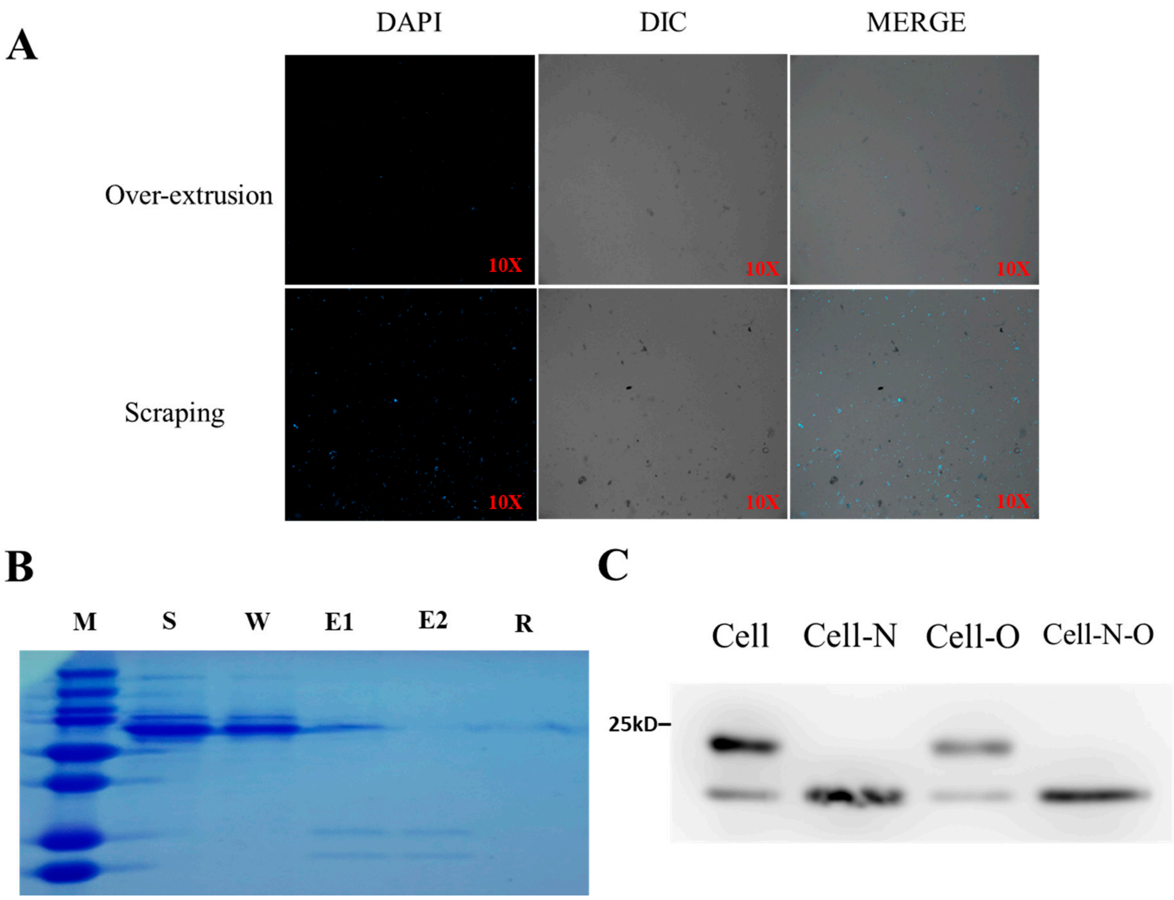

Figure S4. The extraction of mucus from loaches and deglycosylation of purified grass carp IFN1 from 293T cells. The fluorescent staining of DAPI of two different approaches for extracting mucus from loaches. (B) Purification of recombinant IFN1. M: protein marker; S: supernatant; W: washing liquid; E1: first elution; E2: second elution; R: remaining liquid in the column. (C) The deglycosylation of purified grass carp IFN1 from 293T cells. Cell: purified grass carp IFN1 from 293T cells; Cell-N: de-N-glycosylated purified grass carp IFN1 from 293T cells; Cell-O: de-O-glycosylated purified grass carp IFN1 from 293T cells; Cell-N-O: de-N&O-glycosylated purified grass carp IFN1 from 293T cells.

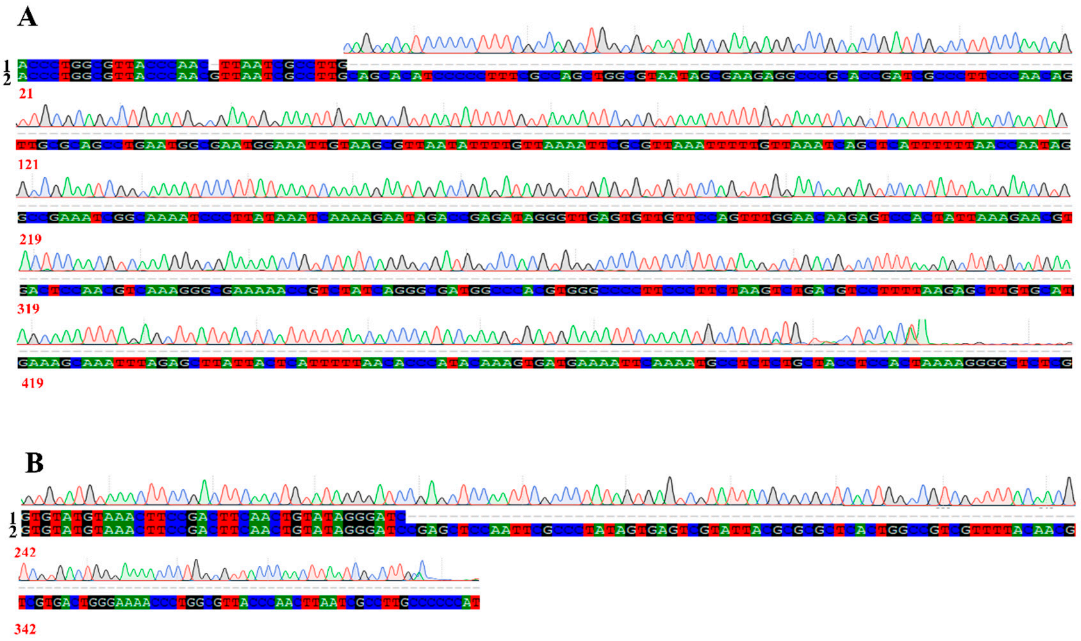

Figure S5. The sequencing data of genome walking from transgenic loaches. (A) The sequencing results of L-terminal; (B) The sequencing results of R-terminal. 1- the sequence of the transgene; 2- the sequence of a genome walking.
